# Septic Pulmonary Embolism Causing Recurrent Pneumothorax in an Intravenous Drug User without Right-Sided Valvular Vegetation in Infective Endocarditis

**DOI:** 10.1155/2021/7050775

**Published:** 2021-11-20

**Authors:** Mason Montano, Kevin Lee, Kushal Patel, Mutsumi Kioka

**Affiliations:** ^1^University of Nevada, Las Vegas, Kirk Kerkorian School of Medicine, 2040 W. Charleston Blvd., 3rd Floor, Las Vegas, NV 89102, USA; ^2^University of Nevada, Las Vegas, Kirk Kerkorian School of Medicine, Department of Critical Care and Pulmonary Medicine, 1701 W. Charleston Blvd., Suite 230, Las Vegas, NV 89012, USA

## Abstract

The following report illustrates a case of a 36-year-old Caucasian male with intravenous drug use (IVDU) induced septic thrombophlebitis presenting with recurrent unilateral pneumothoraces from septic pulmonary embolism (SPE) without the presence of obvious right-sided valvular vegetation in infective endocarditis (IE), defined as tricuspid or pulmonary valve lesions. Pneumothorax (PTX) has been observed as a rare complication of SPE and is commonly associated with infective right-sided IE, IVDU, and intravascular indwelling catheters. However, this case is novel as it is the very rare documented case of recurrent, unilateral, spontaneous right PTX refractory to multiple chest tube placements in such a setting. Therefore, the absence of detectable right-sided valvular vegetation in IE does not obviate the risk of SPE-induced PTX in IVDU and further expands the realm of infectious and pulmonary consequences of SPE and IVDU.

## 1. Introduction

Septic pulmonary embolism (SPE) is an uncommon phenomenon caused by an infected thrombus traveling to the pulmonary vasculature causing a pulmonary embolism, focal lung abscesses, and in some cases pneumothorax (PTX) [1]. The etiology of SPE can be multitudinous, but not limited to intravenous drug use (IVDU), intravascular indwelling catheters, infective endocarditis (IE), liver abscesses, skin and soft tissue infections, septic thrombophlebitis, Lemierre's syndrome, dental infections, and cardiac pacemakers [1]. However, among this exhaustive list, infective endocarditis (IE) is the most common cause of SPE-induced PTX [2]. The following illustrates a novel case of a male with IVDU-induced septic thrombophlebitis presenting with recurrent unilateral pneumothoraces from SPE without the presence of right-sided valvular vegetation.

## 2. Case Presentation

**Figure 1:**
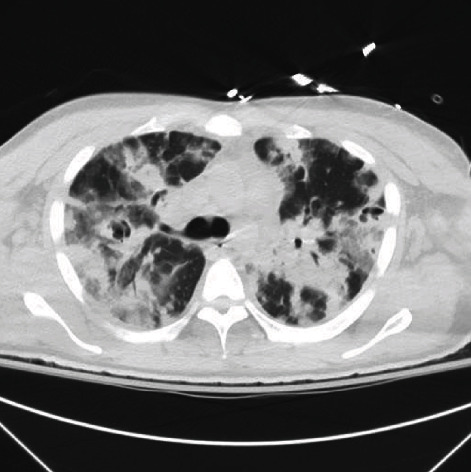
Initial CT chest without contrast findings.

A 36-year-old HIV-negative male with a history of methamphetamine and intravenous heroin use initially presented to an outside facility with left upper extremity cellulitis and dyspnea one week prior to admission. At the outside facility, a computed tomography (CT) of the left upper extremity revealed cellulitic changes with concerns for gas formation. He was transferred due to the need for a higher level of care. Upon arrival, a physical exam revealed the patient in mild distress with bilateral rhonchial breath sounds, extensive edema, and erythema of the left upper extremity without crepitus. Initial laboratory analysis was remarkable for WBC 15.07 k/mm^3^, a urine drug screen positive for amphetamines and opiates, and methicillin-resistant *Staphylococcus aureus* growth in the blood cultures. Repeat CT of the left upper extremity revealed soft tissue swelling without gaseous formations. Chest X-ray revealed bilateral diffuse infiltrates. Intravenous vancomycin, clindamycin, and piperacillin-tazobactam were initiated. However, due to increasing respiratory distress and acute hypoxia to 88% via pulse oximetry, the patient was intubated and placed on ventilator support. A CT chest without contrast revealed diffuse consolidative processes, cavitation foci, diffuse mediastinal and axillary lymphadenopathy, pleural effusions, and areas concerning for septic emboli (see Figure 1).

**Figure 2:**
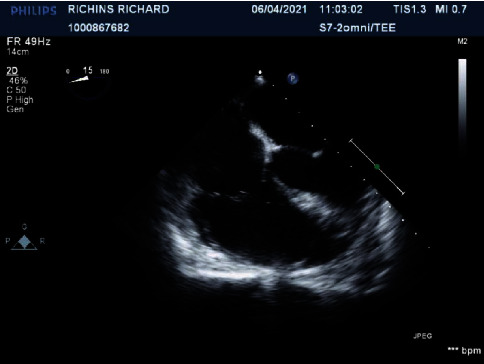
Transesophageal echocardiogram results.

Orthopedic surgery was consulted for debridement of the left upper extremity; however, debridement was delayed due to patient instability. Transthoracic echocardiogram revealed no valvular vegetations. A transesophageal echocardiogram was performed that revealed no valvular abnormalities as well (see Figure 2). Upon culture and sensitivity results, the antibiotics were changed to ceftaroline, gentamicin, and linezolid for presumed IE according to Duke's criteria in the absence of valvular vegetations.

**Figure 3:**
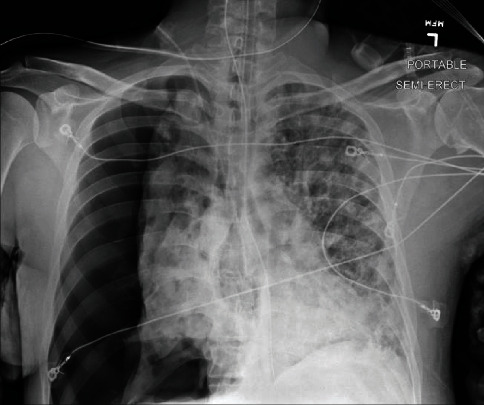
Initial right-sided pneumothorax.

**Figure 4:**
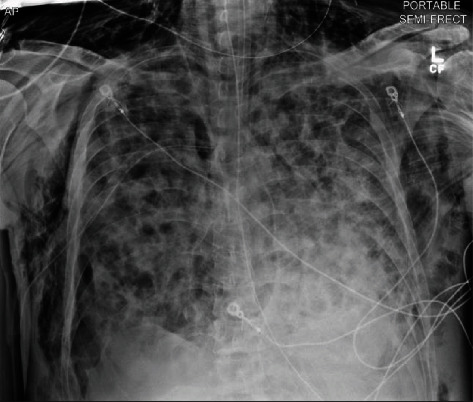
Panthoracic subcutaneous emphysema with single chest tube in place s/p resolution of recurrent PTX.

In the following days, the patient had a secondary spontaneous PTX (see Figure 3) requiring surgical chest tube placement, paralytic usage in addition to renal replacement therapy due to worsening acidosis and uremia. Following the initial placement of the surgical chest tube, a subsequent PTX (see Figure 3) occurred, requiring an additional surgical chest tube to be placed with the resolution of the PTX. Two days following the removal of the first chest tube, the patient spontaneously had the development of large subcutaneous emphysema spreading of the anterior thorax that was improved with increasing the wall suction of the existing chest tube (see Figure 4). After a course of hemodialysis, lung-protective ventilation, neuromuscular blockade, and antibiotic management, a tracheostomy and percutaneous endoscopic gastrostomy were performed, and the patient was downgraded from the intensive care unit to a step-down unit with chest tubes to water seal. The patient was subsequently discharged to a rehabilitation facility.

## 3. Discussion

SPE causing PTX is an exceedingly uncommon pulmonary manifestation of right-sided IE that can have an expansive list of etiologies outside of IE. While IVDU, from heroin or amphetamines, has been illustrated to cause lung parenchymal damage, the infectious complications are typically much more disastrous, as is illustrated and this case report [3], as alluded to IVDU can have numerous infectious and noninfectious pulmonary complications that are not solely limited to SPE and can include PTX [1, 3, 4]. Of these varied complications, there can be thin-walled cavitary lung lesions that guise as pneumatoceles which can further precipitate PTX [1].

PTX secondary to SPE from right-sided IE with valvular vegetations has been previously described by Kappor et al. [2], Aguado et al. [4], and Sheu et al. [5]. This case describes a case of PTX caused by SPE without the presence of right-sided valvular vegetation in IE. According to the Modified Duke Criteria for Infective Endocarditis [6], this patient, even without definite valvular abnormalities or vegetations, falls in the “definite” category for IE and therefore should receive aggressive culture-guided antibiotics. Albeit this is a “definite” case of IE, the absence of valvular abnormalities should not eliminate the suspicion for severe pulmonary sequelae such as SPE and subsequent PTX. In summation, the absence of right-sided valvular lesion in IE does not completely obliviate the occurrence of PTX with the concurrence of SPE and should be on the wide differential in patients who present with IVDU and have a pulmonary compromise.

## Abbreviations

CT: Computed tomography
IVDU: Intravenous drug use
PTX: Pneumothorax
IE: Infective endocarditis
SPE: Septic pulmonary embolism.
